# Impact of Climate Change and Rubber *(Hevea brasiliensis)* Plantation Expansion on Reference Evapotranspiration in Xishuangbanna, Southwest China

**DOI:** 10.3389/fpls.2022.830519

**Published:** 2022-03-03

**Authors:** Zhen Ling, Zhengtao Shi, Shixiang Gu, Tao Wang, Weiwei Zhu, Guojian Feng

**Affiliations:** ^1^Department of Architecture Engineering, Kunming University, Kunming, China; ^2^College of Tourism and Geographic Sciences, Yunnan Normal University, Kunming, China; ^3^State Key Laboratory of Water Resources and Hydropower Engineering Science, Wuhan University, Wuhan, China; ^4^Yunnan Institute of Water & Hydropower Engineering Investigation, Design and Research, Kunming, China

**Keywords:** climate change, rubber plantation, sensitivity coefficient, contribution rate, reference evapotranspiration

## Abstract

The expansion of rubber (*Hevea brasiliensis*) cultivation plantation over the past few decades has been significantly explosive in Xishuangbanna, southwest China. More and more evidences concerning the expansion of rubber plantations lead to the negative influence to local regional hydrology. It is vital to explore the impact of climate change and rubber (*Hevea brasiliensis*) plantation expansion on reference evapotranspiration (ET_0_) for the sustainable and efficient use of regional water resources. In this study, the spatiotemporal variation of ET0 as well as its relationship in rubber plantations area in Xishuangbanna from 1970–2017 were analyzed by using trend, correlation and contribution analysis. The results showed that the rubber plantation was 12,768 ha yr^–1^ from 1990 to 2017 in Xishuangbanna, and nearly 40.8% of new rubber plantations expanded above 900 m in altitude from 2000 to 2017. Sunshine duration and average relative humidity were the key meteorological factors that affect ET_0_ in Xishuangbanna, with the sensitivity coefficient of 0.51 and 0.35, respectively. The multiyear relative change of ET_0_ in Xishuangbanna was 9.18%, and the total contribution of major climate factors was 7.87% during 1970 and 2017. The average relative humidity in the plantation area decreases, which directly leads to the increase of ET_0_. The amount of ET_0_ change from 2000 to 2017 affected by climate change increased at 3.13 mm/10a, whereas it was 2.17 mm/10a affected by the expansion of rubber plantations by quantitative separation. ET_0_ was significantly affected by climate change but intensified by the expansion of rubber plantation.

## Introduction

Evapotranspiration (ET) is one of the most important elements in the water cycle of ecosystem and a key factor in the regional water balance. Reference evapotranspiration (ET_0_) is considered as a crucial factor in hydrological and climate research. The detail of ET_0_ has an important practical significance for analyzing the influence of climate and rubber plantation expansion on regional water resources planning, agricultural planting structure adjustment, and ecological environment protection (Jiao et al., 2020; Xiang et al., 2020; Yang et al., 2021).

Climate and land use and cover change (LUCC) have a strong influence on ET_0_ (Odongo et al., 2019; Zhang et al., 2019; Hu et al., 2021). However, few studies attempted to assess the impact of climate change and commercial agricultural activities such as deforestation. Deforestation is considered as an important anthropogenic process affecting climate and hydrology.

Natural forests and agricultural farming have given ways to commercial agriculture in many areas (Fox, 2012), often in the form of tree plantations such as rubber. Driving by rubber price, expansion rubber plantations have been exploded. Rubber is likely to continue to be one of the fastest-growing land cover types in the world in the coming decades because of their increasing product demand (Warren-Thomas et al., 2015). Furthermore, rubber plantations that expand from traditional areas to uplands with serious negative impacts on the local hydrological cycle cause its high water demand (Tan et al., 2011; Ling et al., 2021).

Meteorological variables such as temperature, wind speed, rainfall, and solar radiation lead to changes in ET_0_. Changes in ET_0_ under climate change depend on these meteorological variables and their interactions.

Decreases in sunshine duration, average relative humidity, wind speed, and saturated water vapor pressure can lead to decreases in ET_0_ (Abtew et al., 2015; Wang et al., 2017). The increase in temperature would lead to the decrease of ET (Fu et al., 2009; Zhang et al., 2013; Pan et al., 2019).

However, changes in meteorological variables are not evenly distributed on the Earth. Globally, for example, RH increased by 0.5%–2.0% per decade over most of the United States, India, and China during the period 1976–2004. There is significant increase in ETo at cold and dry steppe sites (Ghafouri-Azar et al., 2018; Ahmad et al., 2019; D’Andrea et al., 2019; Hwang et al., 2019; You et al., 2019), whereas ET_0_ decreases in humid regions such as Iran and most parts of China (Xu et al., 2018; Nouri and Bannayan, 2019) which include the Yangtze River Delta (Xu et al., 2017). Despite the significant impact of ET_0_ on water resources and ecology, there is very limited knowledge on ET_0_ changes and causes of changes in the tropics area (Pour et al., 2020).

Chen and Huo (2016) used a demeteorological factor trend method to assess the impact of climate change on ET_0_ in the Heihe River Basin. Hu et al. (2021) proved that the expansion of rice area because of human activities was an important factor influencing the change in ET_0_ by trend, correlation, and contribution analyses. Some studies have been conducted on the trends and effects of ET_0_ in southwest China. For instance, sunshine duration, wind speed, and relative humidity are the main factors influencing ET of reference crops in southwest China. ET_0_ increases in spring, autumn, and winter because relative humidity decreases, and ET_0_ decreases in summer because sunshine duration decreases (Zhang Q. W. et al., 2016). However, the influence of climate on ET_0_ in rubber plantation areas is not clear.

Xishuangbanna, an important rubber plantation area in the southwest China since 1950s. Driven by rising prices, the expansion of rubber plantations led to dramatic changes in land use and cover change and they were expanded to higher altitude mountains resulting in a dramatic reduction and fragmentation of natural forest area. Furthermore, rubber plantation expansions caused changes in regional microclimate such as temperature increase, humidity decrease, rainfall decrease, and drought (Gong and Ling, 1996; Qiu, 2009; Lin et al., 2016; Ma et al., 2019; Chiarelli et al., 2020).

In general, there are few studies on the effects of afforestation and other human activities on ET_0_, especially in areas of large expansions of rubber forests. There is a need to systematically explore the effects of increasing rubber forest plantation areas on ET_0_.

Most of the available studies on spatial and temporal changes in ET_0_ of southwest China have focused on trend studies (Shen et al., 2017; Liu et al., 2018) with quantitative analysis of sensitivity coefficients and contributions of meteorological factors to ET_0_. However, few studies separate the effects of climate change and rubber plantation expansion on regional ET_0_.

It is important to figure out the impact of climate change under natural conditions and the expansion of rubber forest area caused by human activities on regional ET_0_ changes.

Thus, we took Xishuangbanna as the study area to conduct these studies: (1) to identify and extract rubber plantations information from 1990 to 2017 using Landsat TM/ETM/OLI images as the basic remote sensing data to analyze the spatial and temporal expansion pattern of rubber plantation; (2) to calculate ET_0_ to combine the daily meteorological observation data from 1970 to 2017 at each meteorological station in the study area with Penman–Monteith formula. We also use sensitivity and contribution analysis to determine the main meteorological influencing factors affected ET_0_ in Xishuangbanna; and (3) to isolate the effects of climate change and rubber plantation expansion on regional ET_0_ changes after compared with the control area not affected by rubber expansion planting.

## Materials and Methods

### Study Area

**FIGURE 1:**
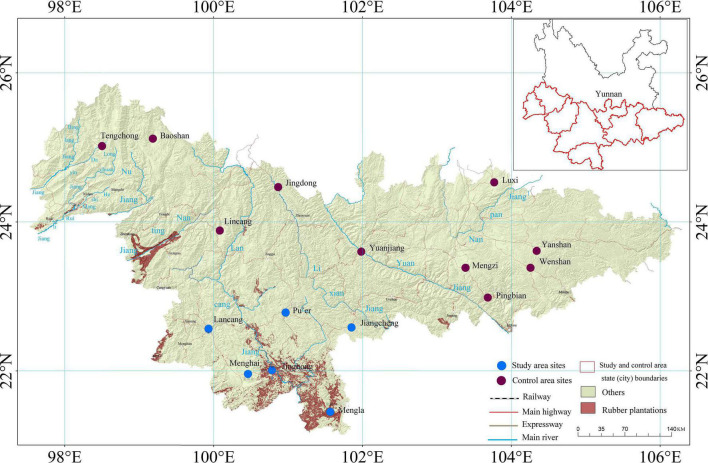
Map of National Meteorological Stations in study area and control area.

Xishuangbanna is located in the southwest China, Yunnan Province (N 21°10′−22°40′, E 99°55′−101°50′). Its total area is 1.97 × 10^4^ km^2^ including 3 prefecture-level cities: Menghai, Mengla, and Jinghong (Zhou et al., 2021). Xishuangbanna is located in the longitudinal valley with a mountainous area of more than 95% and is a low-latitude mountainous area which is controlled and influenced by the warm and humid monsoon with humid, high temperature, and calm wind (Zhang, 1963), as shown in Figure 1.

The temperature is 18°C–22°C, with an annual average temperature of 21.5°C. The annual average duration of sunshine is 1,853.4 h, and the annual average precipitation is 1,599.5 mm. The average relative humidity is about 86% (Ling, 2021).

### Data

Landsat TM/ETM/OLI images were used as the base remote sensing data. The data source was USGS data platform^1^ with a spatial resolution of 30 m by containing multiple spectral bands. The Landsat data level 1 products were used after geometrically corrected and preprocessed using ENVI 5.5 software. The elevation DEM data from the geospatial data cloud platform (see text footnote 1), spatial resolution is 30 m, mainly used to extract slope, slope direction and elevation.

The meteorological data include (1) the daily meteorological data from 1970 to 2017 of 6 typical meteorological stations including Menghai, Mengla, Jinghong, Lancang, Jiangcheng, and Pu’er in rubber plantation areas of Xishuangbanna; (2) the daily meteorological data from 1970 to 2017 from 10 typical stations in southwest Yunnan, including Jingdong, Yuanjiang, Lincang, Tengchong, Baoshan, Pingbian, Mengzi, Wenshan, Yanshan, and Luxi which share the same climate control area, as shown in Table 1.

**TABLE 1:** Information of national meteorological stations in study area and control area.

	Station Code	Station Name	Latitude (°N)	Longitude (°E)	Altitude (m)
Study area	56958	Menghai	100.42	21.92	1177.5
	56969	Mengla	101.34	21.29	631.9
	56959	Jinghong	100.47	22.00	582.2
	56954	Lancang	99.56	22.34	1054.8
	56964	Pu’er	100.58	22.47	1302.1
	56977	Jiangcheng	101.51	22.35	1119.5
Control area	56856	Jingdong	100.87	24.47	1162.3
	56966	Yuanjiang	101.98	23.60	400.9
	56951	Lincang	100.08	23.88	1502.4
	56739	Tengchong	98.50	25.02	1654.6
	56748	Baoshan	99.18	25.12	1652.2
	56986	Pingbian	103.68	22.98	1414.1
	56985	Mengzi	103.38	23.38	1300.7
	56994	Wenshan	104.25	23.38	1271.6
	56991	Yanshan	104.34	23.61	1561.1
	56886	Luxi	103.77	24.53	1704.3

### Methods

#### Identification and Extraction of Rubber Plantation

**FIGURE 2:**
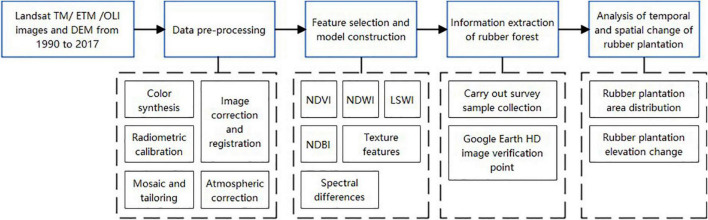
Identification and extraction scheme of rubber plantation.

We use the object-oriented classification method to extract rubber plantations in a hierarchy of typical time windows from February to March by combining the topography of Xishuangbanna and rubber plantation phenology features by ArcGIS 10.2 (Liang et al., 2015; Liu et al., 2017). The remote sensing data on February 22, 1990, February 25, 2000, February 22, 2010, and February 27, 2017 were selected to extract the information of rubber plantation in Xishuangbanna to obtain its spatial distribution in the corresponding years. The detailed scheme is shown in Figure 2.

#### The Calculation of ET_0_

The FAO-56 Penman–Monteith equation (Allen et al., 1998) is as follows:


(1) $$E\,T_{0}=\frac{0.408\,\Delta \,\left(R_{n}-G\right)+\gamma \,\frac{900}{T+273}\,u_{2}\,\left(e_{s}-e_{a}\right)}{\Delta +\gamma \,\left(1+0.34\,u_{2}\right)}$$

where *ET*_0_ is the reference evapotranspiration, mm/d; *G* is the soil heat flux in the study area, MJ/m^2^⋅day; *R*_*n*_ is the net canopy surface radiation, MJ/m^2^⋅day; *u*_2_ is the mean wind speed at 2m, m/s; T is the mean air temperature, °C; *e*_*a*_ is the actual water vapor pressure, kPa; *e*_*s*_ is the saturated water vapor pressure, kPa; γ is the hygrometer constant, kPa/°C; and Δ is the saturated water vapor pressure-temperature slope, kPa/°C.

#### Line Trend Analysis and Manne-Kendall Test

The linear regression model is used to estimate the trend of time series change. The fitting equation is (Ye et al., 2014) as follows:


(2) =yat+b

where *y* is the corresponding value of *ET*_0_ (or season) and other climatic factors; *a* is the tendency rate of the trend, and the positive (or negative) value of *a* represents the increasing (or decreasing) trend of variable *y*; *b* is intercept; and *t* is time series (years). Significance of *ET*_0_ trend assessed using nonparametric M-K test (Li et al., 2016; Feng et al., 2017; Fu, 2018).

Combined with M-K trend method for analysis (Yavuz, 2018):


(3) $$Z=\left\{ \begin{array}{c} \frac{S-1}{\sqrt{V\,a\,r\,\left(S\right)}}\,\begin{array}{cc} & S>0 \end{array}\\ 0\,\begin{array}{ccc} & & S=0 \end{array}\\ \frac{S+1}{\sqrt{V\,a\,r\,\left(S\right)}}\,\begin{array}{cc} & S<0 \end{array} \end{array}\right.$$

(4) $$S=\sum_{i=1}^{n-1}\sum_{j=i+1}^{n}s\,g\,n\,\left(E\,T_{j}-E\,T_{i}\right)$$

(5) $$s\,g\,n\,\left(E\,T_{j}-E\,T_{i}\right)=\left\{ \begin{array}{c} 1\\ 0\\ -1 \end{array}\right.\,\begin{array}{cc} & \begin{array}{c} E\,T_{j}-E\,T_{i}>0\\ E\,T_{j}-E\,T_{i}=0\\ E\,T_{j}-E\,T_{i}<0 \end{array} \end{array}$$

(6) $$v\,a\,r\,\left(S\right)=\frac{n\,\left(n-1\right)\,\left(2\,n+5\right)}{18}$$

where *ET*_*j*_ is the value of *j^th^* data, sgn is the defined sign function; *n* is the length of the time series, when *n* ≥ 8, the statistic *S* approximately obeys normal distribution (Mann, 1945; Kendall, 1975), *Var(S)* is the variance of the statistic *S*; the significance of the trend is tested at α significance level; when the confidence level α = 0.05, 0.1, that is | Z| > 1.96, 1.645, it means that the time series is significant and weakly significant, respectively; when | Z| < 1.645, it means that the time series change is not significant.


(7) $$U\,F=\frac{S-E\,\left(S\right)}{V\,a\,r\,\left(S_{k}\right)}$$

The statistics constitute the positive time series *UF* curve. Based on the same steps, the inverse series statistic is obtained using the time series reversal sample (*k = n,n-1,…,1*), when the two curves *UF* and *UB* intersect within the confidence interval. The intersection point is a mutation point if the *UF* statistic is outside of the confidence interval (with 95% confidence level).

#### Sensitivity Analysis and Contribution Rate of Meteorological Factors

The influence of meteorological factors on *ET*_0_ can be quantified by sensitivity (McCuen, 1974; Beven, 1979; Goyal, 2004; Jia et al., 2016):


(8) $$S_{V\,i}=\lim\left(\frac{\Delta \,E\,T_{0}/E\,T_{0}}{\Delta \,V_{i}/V_{i}}\right)=\frac{\partial E\,T_{0}}{\partial V_{i}}\times \frac{V_{i}}{E\,T_{0}}$$

where *SV*_*i*_ is the sensitivity coefficient; *V*_*i*_ is a meteorological element; *± SV_*i*_* indicates that *ET*_0_ increases (or decreases) as *V*_*i*_ value increases; and *—S_*Vi*_—* indicates the sensitivity of *V*_*i*_ to *ET*_0_.

Contribution of meteorological element *V*_*i*_ to *ET*_0_ change (Yin et al., 2010, 2020):


(9) $$C\,o\,n_{V\,i}=S_{V\,i}\cdot R\,C_{V\,i}$$

(10) $$R\,C_{V\,i}=\frac{n\cdot T\,r\,e\,n\,d}{\left|a\,v\right|}\times 100\%$$

where *Con*_*Vi*_ is the contribution of meteorological element *V*_*i*_ to the change of ET_0_, %; *RC*_*Vi*_ is the multiyear relative change of *V*_*i*_, %; *av* is the average value of meteorological factor *V*_*i*_ for many years; *n* is time series, year; trend is the annual change rate of meteorological factor *V*_*i*_.

The total contribution of each meteorological factor to the change of *ET*_0_ is as follows:


(11) $$C\,o\,n_{E\,T_{0}}=C\,o\,n_{T\,A}+C\,o\,n_{R\,H}+C\,o\,n_{S\,D}+C\,o\,n_{W\,S}$$

where *Con*_*ET0*_ is the total contribution of meteorological elements to ET_0_; *Con*_*TA*_, *Con_*WS*_, Con_*RH*_*, and *Con*_*SD*_ represent the contribution of air temperature, wind speed, average relative humidity, and solar radiation to ET_0_ change.

#### Assessment of ET_0_ Change in Rubber Plantation Expansion

To quantify the contribution of climate change and rubber plantation expansion to ET_0_, the following analyses were conducted in the study and control areas:

(1) We propose a hypothesis that the area of rubber plantations in the study area did not change during this period, and then, any change in ET_0_ in the area is caused by climate change, as ΔET_0_ = ΔET_*oclimate*_ and ΔET_0rubber_ = 0; (2)We assume that the area under rubber plantations in the study area did not change during this period, and then, any change in ET_0_ in the area is caused by climate change.

Therefore, since the impact of climate change on ET_0_ in a region is the same, the impact of rubber plantation expansion on ET_0_ in that region can be identified quantitatively by eliminating the impact of climate change on ET_0_ in that region (Li, 2017; Li et al., 2017; Hu et al., 2021).


(12) $$\Delta \,E\,T_{0}=E\,T_{0}-E\,T_{0\,s}$$

(13) $$\Delta \,E\,T_{0}=\Delta \,E\,T_{0\,c\,l\,i\,m\,a\,t\,e}+\Delta \,E\,T_{0\,r\,u\,b\,b\,e\,r}$$

where Δ*ET_0_* is the annual change of *ET*_0_; Δ*ET_0s_* is stationary *ET*_0_ (recalculated using the detrended data series); Δ*ET_0__*climate*_* and Δ*ET_0__*rubber*_* are the change of *ET*_0_ affected by climate change and expansion of rubber plantation, respectively.

## Results and Analysis

### Changes of Rubber Plantation Area in Xishuangbanna

**FIGURE 3:**
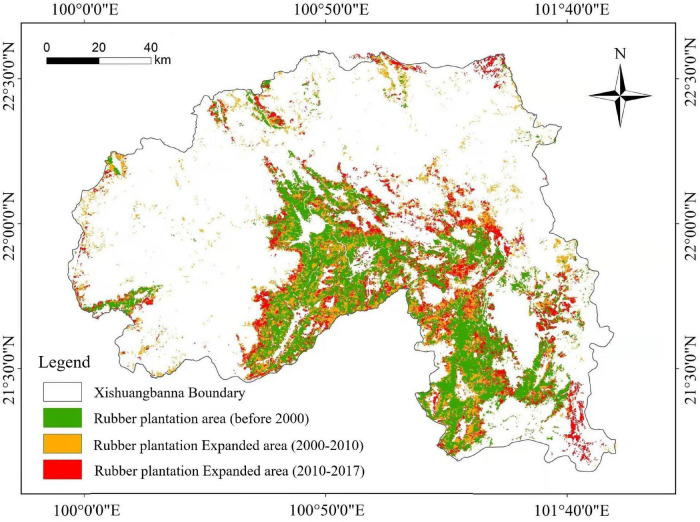
Distribution change of rubber plantations in Xishuangbanna.

The accuracy of land cover categories from 1990 to 2017 was 87.2% and the Kappa coefficient was 0.85. The distribution of rubber plantations in Xishuangbanna from 1990 to 2017 was plotted according to the optimal partition scale of 100, shape index of 0.6, and tightness index of 0.9 in Xishuangbanna, as shown in Figure 3. The rubber plantation area was 121,164 ha in 1990, increased to 211,357 ha in 2000, 404,552 ha in 2010, and reached 465,904 ha in 2017, which accounts for 6.38%, 11.12%, 21.29%, and 24.52% of the land area in Xishuangbanna, respectively.

**FIGURE 4:**
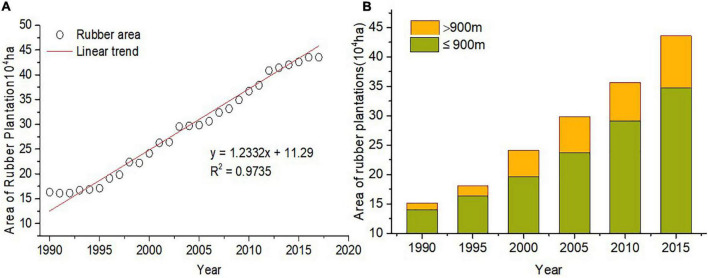
Distribution of rubber plantation area **(A)** and altitude **(B)** in Xishuangbanna.

Rubber plantation was 12,768 ha yr^–1^ from 1990 to 2017 in Xishuangbanna. The growth rate was the largest from 2005 to 2010 when the plantation areas were expanded to uplands. About 87.43% of new rubber plantations were below 900 m in altitude from 1990 to 2000. Nevertheless, there are nearly 40.8% of new rubber plantations shifted to above 900 m in altitude from 2000 to 2017, as is shown in Figure 4.

### Spatiotemporal Changes in ET_0_

**FIGURE 5:**
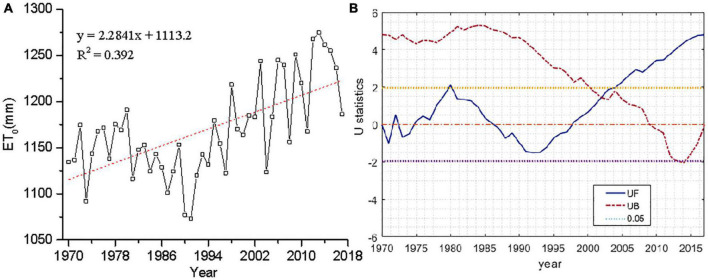
Interannual variation **(A)** and Manne-Kendall (M-K) mutation analysis **(B)** of ET_0_ in Xishuangbanna.

Analyzing the ET_0_ at 6 typical meteorological stations in Xishuangbanna from 1970 to 2017, the maximum value was 1,274.66 mm, the minimum value was 1,073.34 mm, and the average value was 1,169.19 mm. The maximum daily ET_0_ in Xishuangbanna was about 3.5 mm/day. The ET_0_ of each site showed an increasing trend by M-K test, as shown in Figure 5.

The interannual variation trend of ET_0_ in Xishuangbanna increased with a trend of 22.84 mm/10a from 1970 to 2017. The abrupt year of ET_0_ in Xishuangbanna was 2002 by M-K test. It indicates a significant upward change of ET_0_ in 2002, as shown in Table 2.

**TABLE 2:** Interannual variation analysis of ET_0_ at each station in Xishuangbanna.

ET_0_	Maximum value (mm yr^–1^)	Minimum Value (mm yr^–1^)	Mean Value (mm yr^–1^)	Slop	Correlation Index	M-K Abrupt Year
Menghai	1220.78	985.24	1109.37	1.75	0.193*	2002
Mengla	1218.35	1026.99	1119.89	2.29	0.513**	2001
Jinghong	1129.76	935.92	1023.83	0.99	0.101	2005
Jiangcheng	1313.26	1078.44	1185.56	2.60	0.436**	1998
Pu’er	1206.69	972.04	1082.04	2.59	0.426**	1998
Lancang	1341.99	1113.71	1212.64	2.50	0.418**	1998

*Significance at 0.05; **Significance at 0.01.

**FIGURE 6:**
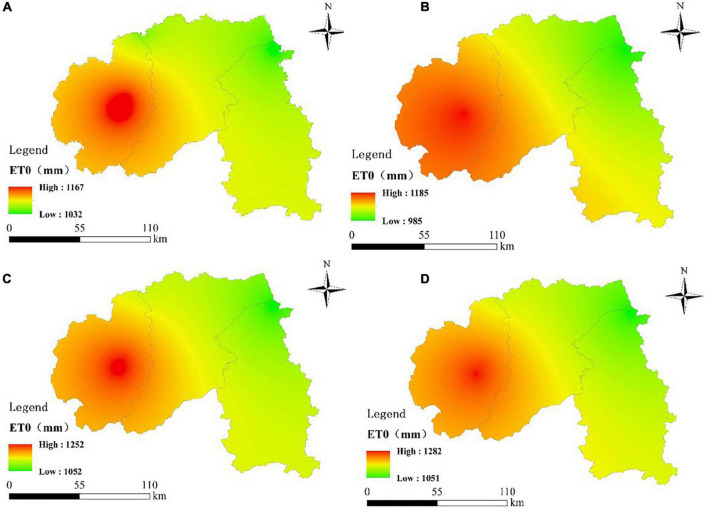
Average ET_0_ during **(A)** 1970–1990, **(B)** 1991–2000, **(C)** 2001–2010, and **(D)** 2011–2017.

The spatial distribution of ET_0_ in Xishuangbanna from 1970 to 2017 is shown in Figure 6. The distribution is different in each period. The areas with high ET_0_ value mainly concentrated in Menghai County in the southwest, which shows high in the southwest and low in the northeast. The range of ET_0_ changes in Xishuangbanna did not change much in 1970–1990 and 1991–2000 (Figures 6A,B), while the ET_0_ values increased significantly and the area of high ET_0_ value expanded after 2000 (Figures 6C,D). The ET_0_ in Xishuangbanna from 1970 to 2017 showed an increasing trend. Meanwhile, the high ET_0_ area gradually increased.

### Characterization of Meteorological Elements in Rubber Plantation Areas in Xishuangbanna

The meteorological elements in the planting area of Xishuangbanna region from 1970 to 2017 are shown in Table 3. Precipitation in Xishuangbanna decreased at a rate of 0.018mm yr^–1^. Jinghong had the most obvious precipitation reduction. Sunshine duration increased at a rate of 0.012h d^–1^yr^–1^, and the average relative humidity changed at a rate of 0.258%yr^–1^. Xishuangbanna is a calm wind area with a slight increase in the change rate of wind speed. The average change rate of wind speed is 0.004 m s^–1^ yr^–1^. The maximum temperature in Xishuangbanna increased at a rate of 0.029°C yr^–1^. Menghai was the area with the most significant increase in maximum temperature. The lowest temperature increased at a rate of 0.042°C yr^–1^, and Pu’er is the most obviously lowest temperature increased. Temperature, sunshine duration, and average wind speed increased, whereas average relative humidity and precipitation decreased in Xishuangbanna from 1970 to 2017, as shown in Table 3.

**TABLE 3:** Interannual variation trend of meteorological elements at each meteorological station.

Station Name	Precipitation (mm yr^–1^)	Sunshine Duration (h d^–1^yr^–1^)	Average Relative Humidity (% yr^–1^)	Average Wind Speed (m s^–1^ yr^–1^)	Maximum Temperature (°C yr^–1^)	Minimum Temperature (°C yr^–1^)
Menghai	−0.005	0.016	−0.120	0.005	0.035	0.051
Mengla	−0.015	0.011	−0.959	0.012	0.030	0.031
Jinghong	−0.034	0.015	−0.137	0.012	0.033	0.030
Jiangcheng	−0.019	−0.00003	−0.089	−0.009	0.028	0.039
Pu’er	−0.019	0.013	−0.162	−0.002	0.030	0.061
Lancang	−0.014	0.017	−0.083	0.006	0.017	0.041
Mean	−0.018	0.012	−0.258	0.004	0.029	0.042

### Attribution Analysis of ET_0_ Change of in Xishuangbanna

#### Correlation Analysis Between ET_0_ of Meteorological Stations and Annual Mean Meteorological Elements

According to formula (1), we selected six meteorological factors for correlation analysis with the annual ET_0_ in the rubber plantation area of Xishuangbanna, as shown in Table 4.

**TABLE 4:** Linear correlation coefficient between ET_0_ of each station and annual mean meteorological elements.

Sites	Maximum Temperature	Minimum Temperature	Average Relative Humidity	Wind Speed	Sunshine Duration	Precipitation
Menghai	0.35*	0.49**	−0.55**	0.49**	0.69**	−0.29*
Mengla	0.47**	0.20	−0.52**	0.15	0.86**	−0.02
Jinghong	0.62**	0.42**	−0.57**	0.59**	0.09	−0.07*
Jiangcheng	0.49**	0.34*	−0.52**	−0.19	0.37*	−0.15
Pu’er	0.73**	0.77**	−0.88**	−0.13	0.71**	−0.37*
Lancang	0.51**	0.32*	−0.64**	0.12	0.59*	−0.08

*Significance at 0.05; **Significance at 0.01.

In the past 47 years, the annual mean ET_0_ in Xishuangbanna was significantly positively correlated with T_*max*_ and sunshine duration, the correlation coefficients of 0.35–0.73 and 0.37–0.86. It was significantly negatively correlated with RH, and the correlation coefficients of 0.52−0.88 (shown in Table 4). In addition to T_*max*_, sunshine duration and average relative humidity in Menghai, Jinghong, and Pu’er were negatively correlated with precipitation with correlation coefficients of 0.29, 0.07, and 0.37, respectively. The ET_0_ decreased because the more precipitation leads to higher average relative humidity and less sunshine duration. Menghai, Jinghong, and Lancang were significantly and positively correlated with wind speed with the correlation coefficients of 0.49, 0.59, and 0.12, respectively. The greater the wind speed, the more increase of ET_0_.

#### Sensitivity Analysis of ET_0_ to Each Meteorological Element

The ET_0_ in Xishuangbanna showed an increasing trend influenced by the changes in each meteorological factor during 1970 and 2017. The extent to ET_0_ affected by changes in meteorological factors was quantified by analyzing individual factor sensitivities (Dong et al., 2019).

The sensitivity coefficients of different station meteorological variables on ET_0_ variation in Xishuangbanna region are shown in Table 5. The effects of temperature, wind speed, and sunshine duration on ET_0_ variation were positive, whereas the effects of average relative humidity on ET_0_ variation were relatively negative. Sunshine duration is the most sensitive meteorological factor for ET_0_ changes in the region with a mean sensitivity coefficient of 0.51. The mean wind speed is 0.09, which is the lowest factor.

**TABLE 5:** Correlation coefficients of ET_0_ and meteorological variables.

Station	Average Temperature	Average Relative Humidity	Sunshine Duration	Wind Speed
Menghai	0.27	−0.32	0.53	0.09
Jinghong	0.34	−0.38	0.51	0.06
Mengla	0.31	−0.36	0.54	0.07
Jiangcheng	0.37	−0.32	0.5	0.11
Pu’er	0.31	−0.35	0.51	0.12
Langcan	0.28	−0.38	0.49	0.11
Mean	0.31	−0.35	0.51	0.09

All the meteorological factors showed positive contributions to the ET_0_ among which sunshine duration had the largest contribution and then followed by average relative humidity, whereas wind speed had the lowest contribution because of its low sensitivity coefficient to ET_0._

As shown in Table 6, the contribution of each meteorological factor to ET_0_ was positive during 47 years in which the contribution of sunshine duration was the highest about 3.41. Then, average relative humidity is 1.65. Wind speed had relative high variations but the low sensitivity coefficient to ET_0_ that the total contribution was 1.34. Totally, the annual relative variation of ET_0_ and the main meteorological factors contributed is 9.18% and 7.87% in Xishuangbanna.

**TABLE 6:** Sensitivity coefficient of meteorological elements in Xishuangbanna, their relative changes over the years and their contributions to ET_0_ change.

Meteorological factor	Sensitivity coefficient	Multiyear relative change (%)	Contribution (%)
Air temperature	0.31	4.76	1.47
Average relative humidity	−0.35	−4.7	1.65
Sunshine duration	0.51	6.68	3.41
Wind speed	0.09	14.84	1.34
ET_0_		9.18	7.87

#### Response of Climate Change and Rubber Plantation Expansion to ET_0_

Average relative humidity and sunshine duration are the key influential meteorological factors that affect ET_0_ in Xishuangbanna by sensitivity and contribution analysis. It can be inferred that rising sunshine duration and decreasing average relative humidity caused the increase of ET_0_ in Xishuangbanna from 1970 to 2017. The increase in sunshine duration promotes evaporation rate whereas the decrease in average relative humidity characterizes the decrease in water vapor content in the subsurface.

A significant trend of increasing ET_0_ in 2000 which was coupled with rubber plantation expansion by M-K analysis of ET_0_ in Xishuangbanna. Hence, the changes in average relative humidity and sunshine duration in Xishuangbanna after 2000 were analyzed to discuss the effects of climate change and rubber plantation expansion on ET_0_.

**FIGURE 7:**
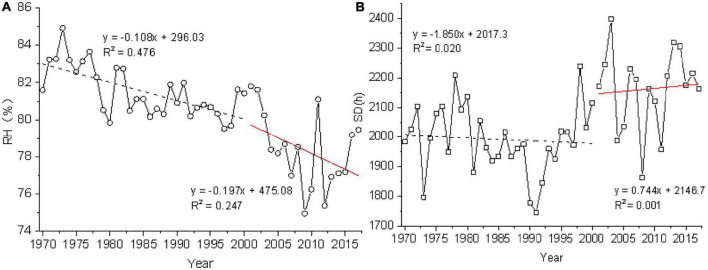
Interannual variation trend of average relative humidity **(A)** and sunshine duration **(B)** in rubber plantation area of Xishuangbanna.

As shown in Figure 7, the average relative humidity in Xishuangbanna for 47 years showed a decreasing trend. During 2000, it was 1.08%/10a from 1970 to 2000 and 1.97%/10a from 2000 to 2017 with a significant decrease trend of 45.18% in average relative humidity. The decreasing trend of sunshine duration averaged 18.5h/10a from 1970 to 2000, which shows an upward trend averaged 7.44h/10a, and an increase trend of 59.78% from 2000 to 2017.

To analyze the influence of climate and rubber plantation expansion on ET_0_ changes in Xishuangbanna. The control areas such as Jingdong, Lincang, Baoshan, and Yanshan in the same climate area without were affected by the wide expansion of rubber plantations for comparative.

**FIGURE 8:**
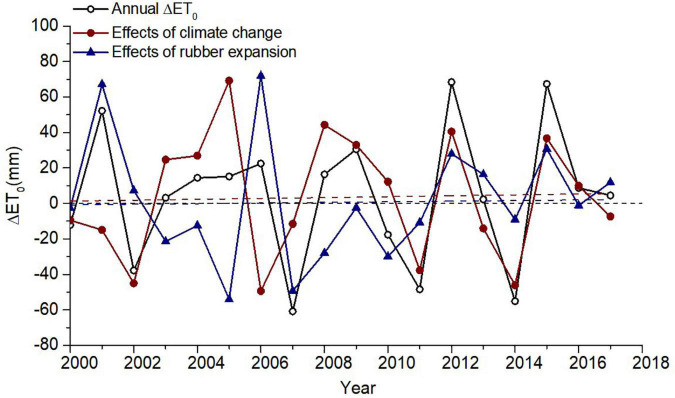
Fluctuation of the impact of climate change and rubber plantation expansion on the annual mean ET0 change in Xishuangbanna.

Calculation of ET_0_ under rubber plantation expansion and climate change using Equations (12) and (13), as shown in Figure 8:

We compared the ET_0_ change from 2000 to 2017, and ET_0_ in the control region increased at a rate of 3.13 mm/10a due to climate change. However, ET_0_ in Xishuangbanna increased at a rate of 2.17 mm/10a due to the expansion of rubber plantation (the effect of climate change on ET_0_ in Xishuangbanna has been removed). The results indicated that even the effect of rubber plantation expansion on ET_0_ in Xishuangbanna from 2000 to 2017 was less than that of climate change, but rubber plantation expansion exacerbated the increase of ET_0_.

However, rubber plantation expansion had a greater impact on ET_0_ than climate change in some periods. For example, rubber plantations in Xishuangbanna showed a sharp growth due to the continuous increase in international rubber prices before 2011 (Chen et al., 2016). After that, the influence of climate change on ET_0_ became gradually dominated after 2011. Generally, the effects of climate change and rubber plantation expansion on ET_0_ in Xishuangbanna during 2000 and 2017 were dynamically changed and coacted.

## Discussion

### ET_0_ Changes by the Influenced of Meteorological Elements

Precipitation showed a decreasing trend especially in Jinghong, Jiangcheng, and Pu’er. The sunshine duration increased at most of stations except Jiangcheng. The average relative humidity decreased. The wind speed showed a less increasing trend. The average maximum and minimum temperature also increased. Zhao et al. (2012) indicated the temperature increase in Xishuangbanna (annual average temperature 0.013°C/a; annual average ground temperature 0.007°C/a); Peng et al. (2020) also proved temperature increase and precipitation decrease in Xishuangbanna from 1961 to 2016.

ET_0_ in Xishuangbanna showed increase from 1970 to 2017 due to the average relative humidity decreased and sunshine duration increased (Zuo et al., 2012; Xie and E, 2014; Abtew et al., 2015). Influenced by the differences in geographical and temporal scales, Kousari and Ahani (2012) found that ET_0_ has an increasing trend in Iran. Chaouche et al. (2010) also proved ET_0_ increased in the Mediterranean region which are consistent with the results of our study. However, Wang et al. (2017) studied 1961–2013 in China with a decreasing ET_0_.

### ET_0_ Changes by the Influenced of Rubber Plantation Expansion

With the close international cooperation and growing demand for rubber trade, the rubber plantation in Xishuangbanna has increased rapidly with 39.68% compared to the pre-2000 period. Indeed, it has been expanded to higher altitude mountain where is the natural forest retaining area (Xu et al., 2014; Chambon et al., 2016; Chen et al., 2016). Natural forest was replaced and the coverage rate decreased from 69.0 to 43.6%.

The dramatic expansion of rubber plantations has altered the local and regional water balance, which led to changes in the regional microclimate. The ET_0_ changes with the microclimate of the area.

Rubber, the native to the Amazon rainforest, has a well-developed root system and xylem conduits. The root-water extraction moves from shallow to deep soil layers as the seasons and soil moisture zone change (Liu et al., 2013, 2014; Zeng et al., 2019).

Compared with secondary forests, rubber trees have higher ET and higher demand for water resources (Lin et al., 2018; Ma et al., 2019; Ling et al., 2021).

The expansion of a single rubber forest plantation has contributed to the continuous reduction of the natural forest area and the intensification of habitat fragmentation, which in turn led to regional soil erosion (Liu, 2015; Guillaume et al., 2016), a decrease in the ability to contain water (Ma et al., 2019; Zou et al., 2020), a decrease in relative air humidity, a decrease in fog days, and a gradual shift in climate from hot and humid to hot and dry, accelerating the formation of climate extremes in Xishuangbanna (Gong and Ling, 1996; Martius et al., 2004; Qiu, 2010; Chen et al., 2016; Peng et al., 2020) and causing negative ecohydrological effects such as a shortage of regional water resources (Guardiola-Claramonte et al., 2008; Ziegler et al., 2009; Zhou et al., 2011).

People live in the rubber plantation area experience a rare water shortage and the rivers seasonal breakdowns (He et al., 2007; Li et al., 2008; Chiarelli et al., 2020; Ling et al., 2021). Xishuangbanna is well-known clam wind and foggy area. Fog precipitation is a very important source of moisture and nutrients. Currently, the fog days decreased, the duration of fog is shorter and fog water content decreased by the impact of human activities in Xishuangbanna (Gong and Ling, 1996; Huang et al., 2001). The decrease of relative humidity leads to the increase of ET_0_.

### Contributions of Climate Change and Rubber Plantation Expansion to ET_0_ Trends

There are significant differences in ET_0_ sensitivity to climate variables in different regions and under different climatic conditions (Zeng et al., 2010; Chen and Huo, 2011). The positive contribution of rising sunshine duration and decreasing average relative humidity in Xishuangbanna caused the increase of ET_0_ over the past 47 years. Lakshman and Gicy (2006) and Yin et al. (2010) studied that the solar radiation in the humid–semihumid zone plays more important role. Sunshine duration is the most important factor that affects ET_0_ in our study area, followed by the average relative humidity and temperature. The results are consistent with Cao et al. (2015) and Zhang W. W. et al. (2016) who found that the dominant factor of ET_0_ variation in mainland and southwest China is sunshine duration. However, temperature is the most sensitive factor to ET_0_ in northeast China whereas solar radiation is most sensitive in northwest China.

Based on assessment of ET_0_ change in rubber plantation expansion, the effects of climate change and rubber plantation expansion on ET_0_ were partitioned. The conclusion that climate change and rubber plantation expansion in the study area have the same influence on ET_0_ changes. Rubber plantation expansion exacerbated the change in ET_0_. Li et al. (2017) quantified the effects of land use and climate change on ET in China.

Climate change was more significant than LUCC change in influencing ET in China during the period 2001 and 2013. Jia et al. (2016) proved that average relative humidity and sunshine duration were sensitive factors for the change of ET_0_. The decrease of sunshine duration led to the decreased ET_0_. The effects of irrigation and climate change on ET_0_ were 49.97% and 50.03%, respectively. Han et al. (2014) suggested that ET_0_ decreased in Jingtai irrigation district because of irrigation change regional climatic factors such as wind speed decreased and average relative humidity increased. Rubber plantation expansion also changes ET_0_ due to changes in regional microclimate.

## Conclusion

In this paper, we analyzed the spatial and temporal characteristics of rubber plantations and ET_0_ in Xishuangbanna, Yunnan Province from 1970 to 2017, and we also analyzed the relationship between ET_0_, rubber plantation area, and climate change. The conclusions are as follows:

(1) The ET_0_ increased with 22.84 mm/10a from 1970 to 2017. The spatial distribution of ET_0_ was higher in the southwest and lower in the northeast, whereas the ET_0_ values increased significantly and the area of high ET_0_ expanded after 2000. Rubber plantation was 12,768 ha yr^–1^ from 1990 to 2017 in Xishuangbanna. The growth rate was the largest from 2005 to 2010 in the plantation areas expanded to uplands.
(2) Average relative humidity and sunshine duration are the key meteorological factors that affect ET_0_ in Xishuangbanna. The multiyear relative change of ET_0_ in Xishuangbanna in 47a was 9.18%, which showed an increasing trend and the total contribution of the main climate factors to it was 7.87%.
(3) Climate change and rubber plantation expansion in Xishuangbanna increased ET_0_. Rubber plantation expansion decreased the average relative humidity which intensified the increase of ET_0_.

Due to the extremely complex mechanisms of land surface–atmosphere interactions, it is difficult to directly distinguish the effects of rubber plantation expansion from climate change on ET_0._ In addition, meteorological station data inevitably bring some errors and uncertainties. Although the independent effects of climate change and rubber plantation expansion on ET_0_ were calculated separately, climate change and rubber plantation expansion interact with each other. The climate change factors influence the boundary of rubber plantation expansion, which also affects the regional climate (Ellison et al., 2012). The interaction between regional climate, rubber plantation expansion, and energy (water) balance needs to be studied by adding more regional surface models to provide more theoretical supporting. However, it can support for the negative hydroecological effects such as local water resources shortage caused by the expansion of rubber plantations.

## Data Availability Statement

The original contributions presented in the study are included in the article/supplementary material, further inquiries can be directed to the corresponding author.

## Author Contributions

ZL: conceptualization, methodology, software, investigation, writing—original draft preparation, and writing—reviewing and editing. ZS: conceptualization, supervision, and writing—reviewing and editing. SG: visualization, software, and investigation. TW: software and validation. WZ: data curation and methodology. GF: software and writing—reviewing and editing. All authors contributed to the article and approved the submitted version.

## Conflict of Interest

The authors declare that the research was conducted in the absence of any commercial or financial relationships that could be construed as a potential conflict of interest.

## Publisher’s Note

All claims expressed in this article are solely those of the authors and do not necessarily represent those of their affiliated organizations, or those of the publisher, the editors and the reviewers. Any product that may be evaluated in this article, or claim that may be made by its manufacturer, is not guaranteed or endorsed by the publisher.

## Footnotes

1
http://glovis.usgs.gov/
