# Corrigendum: Impact of climate change and rubber (*Hevea brasiliensis*) plantation expansion on reference evapotranspiration in Xishuangbanna, Southwest China

**DOI:** 10.3389/fpls.2022.1092168

**Published:** 2023-01-24

**Authors:** Zhen Ling, Zhengtao Shi, Shixiang Gu, Tao Wang, Weiwei Zhu, Guojian Feng

**Affiliations:** ^1^Department of Architecture Engineering, Kunming University, Kunming, China; ^2^Faculty of Geography, Yunnan Normal University, Kunming, China; ^3^State Key Laboratory of Water Resources and Hydropower Engineering Science, Wuhan University, Wuhan, China; ^4^Yunnan Institute of Water & Hydropower Engineering Investigation, Design and Research, Kunming, China

**Keywords:** climate change, rubber plantation, sensitivity coefficient, contribution rate, reference evapotranspiration

In the published article, there was an error in **Table 3**. We made a mistake when we wrote the “Average relative humidity” data of correlation coefficients of ET_0_ as “5.” The corrected **Table 3** appears below.

**Table 3:** Correlation coefficients of ET_0_ and meteorological variables.

Sites	Average temperature	Average relative humidity	Sunshine duration	Wind speed
Menghai	0.27	-0.32	0.53	0.09
Jinghong	0.34	-0.38	0.51	0.06
Mengla	0.31	-0.36	0.54	0.07
Jiangcheng	0.37	-0.32	0.50	0.11
Puer	0.31	-0.35	0.51	0.12
Langcan	0.28	-0.38	0.49	0.11
Mean value	0.31	-0.35	0.51	0.09

In the published article, **Tables 3**, **4** and **5** were incorrectly numbered. The corrected table numbers are below.

**Table 3:** Interannual variation trend of meteorological elements at each meteorological station.

Station Name	Precipitation (mm yr^-1^)	Sunshine duration (h d^-1^yr^-1^)	Average relative humidity (% yr^-1^)	Average wind speed (m s^-1^ yr^-1^)	Maximum Temperature (°C yr^-1^)	Minimum Temperature (°C yr^-1^)
Menghai	-0.005	0.016	-0.120	0.005	0.035	0.051
Mengla	-0.015	0.011	-0.959	0.012	0.030	0.031
Jinghong	-0.034	0.015	-0.137	0.012	0.033	0.030
Jiangcheng	-0.019	-0.00003	-0.089	-0.009	0.028	0.039
Pu’er	-0.019	0.013	-0.162	-0.002	0.030	0.061
Lancang	-0.014	0.017	-0.083	0.006	0.017	0.041
Mean	-0.018	0.012	-0.258	0.004	0.029	0.042

**Table 4:** Linear correlation coefficient between ET_0_ of each station and annual mean meteorological elements.

Sites	Maximum temperature	Minimum Temperature	Average relative humidity	Wind Speed	Sunshine Duration	Precipitation
Menghai	0.35*	0.49**	-0.55**	0.49**	0.69**	-0.29*
Mengla	0.47**	0.20	-0.52**	0.15	0.86**	-0.02
Jinghong	0.62**	0.42**	-0.57**	0.59**	0.09	-0.07**
Jiangcheng	0.49**	0.34*	-0.52**	-0.19	0.37*	-0.15
Pu’er	0.73**	0.77**	-0.88**	-0.13	0.71**	-0.37*
Lancang	0.51**	0.32*	-0.64**	0.12	0.59^*^	-0.08

*Significance at 0.05; **Significance at 0.01.

**Table 5:** Correlation coefficients of ET_0_ and meteorological variables .

Station	Average Temperature	Average Relative Humidity	Sunshine Duration	Wind Speed
Menghai	0.27	-0.32	0.53	0.09
Jinghong	0.34	-0.38	0.51	0.06
Mengla	0.31	-0.36	0.54	0.07
Jiangcheng	0.37	-0.32	0.5	0.11
Pu’er	0.31	-0.35	0.51	0.12
Langcan	0.28	-0.38	0.49	0.11
Mean	0.31	-0.35	0.51	0.09

In the published article, there was an error in the **Sensitivity Analysis of ET0 to Each Meteorological Element** section, second paragraph. We mixed the data of other research by mistake and the below sentence is removed.

“Wind speed in Xishuangbanna showed the largest change which is followed by sunshine duration, average relative humidity, and air temperature with 11.71%, 4.39%, 4.32%, and 2.84%, respectively.”

In the published article, a table was incorrectly cited in the **Characterization of Meteorological Elements in Rubber Plantation Areas in Xishuangbanna** section, paragraph 1. The corrected sentence appears below:

“Temperature, sunshine duration, and average wind speed increased, whereas average relative humidity and precipitation decreased in Xishuangbanna from 1970 to 2017, as shown in **Table 3**.”

In the published article, a table was incorrectly cited in the **Attribution Analysis of ET_0_ Change of in Xishuangbanna** section, paragraph 1. The corrected sentence appears below:

“According to formula (1), we selected six meteorological factors for correlation analysis with the annual ET_0_ in the rubber plantation area of Xishuangbanna, as shown in **Table 4**.”

In the published article, a table was incorrectly cited in the **Attribution Analysis of ET_0_ Change of in Xishuangbanna** section, paragraph 2. The corrected sentence appears below:

“It was significantly negatively correlated with RH, and the correlation coefficients of 0.52-0.89 (shown in **Table 4**).”

In the published article, a table was incorrectly cited in the **Attribution Analysis of ET_0_ Change of in Xishuangbanna** section, paragraph 2. The corrected sentence appears below:

“The sensitivity coefficients of different station meteorological variables on ET_0_variation in Xishuangbanna region are shown in **Table 5**.”

The authors apologize for these errors and state that this does not change the scientific conclusions of the article in any way. The original article has been updated.

