# Corrigendum: Impact of climate change and rubber (*Hevea brasiliensis*) plantation expansion on reference evapotranspiration in Xishuangbanna, Southwest China

**DOI:** 10.3389/fpls.2023.1157058

**Published:** 2023-03-03

**Authors:** Zhen Ling, Zhengtao Shi, Shixiang Gu, Tao Wang, Weiwei Zhu, Guojian Feng

**Affiliations:** ^1^ Department of Architecture Engineering, Kunming University, Kunming, China; ^2^ College of Tourism and Geographic Sciences, Yunnan Normal University, Kunming, China; ^3^ State Key Laboratory of Water Resources and Hydropower Engineering Science, Wuhan University, Wuhan, China; ^4^ Yunnan Institute of Water & Hydropower Engineering Investigation, Design and Research, Kunming, China

**Keywords:** climate change, rubber plantation, sensitivity coefficient, contribution rate, reference, evapotranspiration

In the published article, **Tables 3**–**5**, **Table 4** should be moved to become **Table 3**, and **Table 5** should be moved to become **Table 4**. There was an error in **Table 3** as published. There was a mistake in the “Average relative humidity” data of correlation coefficients of ET_0_: “5” was written as “3” in the correlation coefficient of “Average relative humidity” in meteorological stations in ET_0_. The corrected tables appear below.

**Table 3:** Interannual variation trend of meteorological elements at each meteorological station.

Station Name	Precipitation (mm yr^−1^)	Sunshine Duration (h d^−1^ yr^−1^)	Average Relative Humidity (% yr^−1^)	Average Wind Speed (m s^−1^ yr^−1^)	Maximum Temperature (°C yr^−1^)	Minimum Temperature (°C yr^−1^)
Menghai	−0.005	0.016	−0.120	0.005	0.035	0.051
Mengla	−0.015	0.011	−0.959	0.012	0.030	0.031
Jinghong	−0.034	0.015	−0.137	0.012	0.033	0.030
Jiangcheng	−0.019	−0.00003	−0.089	−0.009	0.028	0.039
Pu’er	−0.019	0.013	−0.162	−0.002	0.030	0.061
Lancang	−0.014	0.017	−0.083	0.006	0.017	0.041
Mean	−0.018	0.012	−0.258	0.004	0.029	0.042

**Table 4:** Linear correlation coefficient between ET_0_ of each station and annual mean meteorological elements.

Sites	Maximum Temperature	Minimum Temperature	Average Relative Humidity	Wind Speed	Sunshine Duration	Precipitation
Menghai	0.35*	0.49**	−0.55**	0.49**	0.69**	−0.29*
Mengla	0.47**	0.20	−0.52**	0.15	0.86**	−0.02
Jinghong	0.62**	0.42**	−0.57**	0.59**	0.09	−0.07*
Jiangcheng	0.49**	0.34*	−0.52**	−0.19	0.37*	−0.15
Pu’er	0.73**	0.77**	−0.88**	−0.13	0.71**	−0.37*
Lancang	0.51**	0.32*	−0.64**	0.12	0.59^*^	−0.08

*Significance at 0.05; **Significance at 0.01.

**Table 5:** Correlation coefficients of ET_0_ and meteorological variables

Station	Average Temperature	Average Relative Humidity	Sunshine Duration	Wind Speed
Menghai	0.27	−0.32	0.53	0.09
Jinghong	0.34	−0.38	0.51	0.06
Mengla	0.31	−0.36	0.54	0.07
Jiangcheng	0.37	−0.32	0.5	0.11
Pu’er	0.31	−0.35	0.51	0.12
Langcan	0.28	−0.38	0.49	0.11
Mean	0.31	−0.35	0.51	0.09

In the published article, several errors were made in **Results and Analysis** “Attribution Analysis of ET_0_ Change of in Xishuangbanna”. We have deleted the below sentence:

“Wind speed in Xishuangbanna showed the largest change which is followed by sunshine duration, average relative humidity, and air temperature with 11.71%, 4.39%, 4.32%, and 2.84%, respectively.”

We have also corrected the following sentences:

Incorrect sentence:

“According to formula (1), we selected six meteorological factors for correlation analysis with the annual ET_0_ in the rubber plantation area of Xishuangbanna, as shown in **Table 5**.”

The corrected sentence:

“According to formula (1), we selected six meteorological factors for correlation analysis with the annual ET_0_ in the rubber plantation area of Xishuangbanna, as shown in **Table 4**.”

Incorrect sentence:

“It was significantly negatively correlated with RH, and the correlation coefficients of 0.52–0.89 (shown in **Table 5**).”

The corrected sentence:

“It was significantly negatively correlated with RH, and the correlation coefficients of 0.52–0.89 (shown in **Table 4**).”

Incorrect sentence:

“The sensitivity coefficients of different station meteorological variables on ET_0_variation in Xishuangbanna region are shown in **Table 6**.”

**Table 6:** Sensitivity coefficient of meteorological elements in Xishuangbanna, their relative changes over the years and their contributions to ET_0_ change.

Meteorological factor	Sensitivity coefficient	Multiyear relative change (%)	Contribution (%)
Air temperature	0.31	4.76	1.47
Average relative	-0.35	-4.7	1.65
Humidity			
Sunshine duration	0.51	6.68	3.41
Wind speed	0.09	14.84	1.34
ET_0_		9.18	7.87

The corrected sentence:

“The sensitivity coefficients of different station meteorological variables on ET_0_variation in Xishuangbanna region are shown in **Table 5**.”

Incorrect sentence:

“It was significantly negatively correlated with RH, the correlation coefficients of 0.52–0.89 (as shown in **Table 5**). In addition to Tmax, sunshine duration and average relative humidity in Menghai, Jinghong, and Pu’er were negatively correlated with precipitation with correlation coefficients of 0.29, 0.07, and 0.36, respectively. The ET_0_ decreased because the more precipitation leads to higher average relative humidity and less sunshine duration. Menghai, Jinghong, and Lancang were significantly and positively correlated with wind speed with the correlation coefficients of 0.49, 0.59, and 0.37, respectively.”

The corrected sentence:

“It was significantly negatively correlated with RH, the correlation coefficients of 0.52–0.88 (as shown in **Table 4**). In addition to Tmax, sunshine duration and average relative humidity in Menghai, Jinghong, and Pu’er were negatively correlated with precipitation with correlation coefficients of 0.29, 0.07, and 0.37, respectively. The ET_0_ decreased because the more precipitation leads to higher average relative humidity and less sunshine duration. Menghai, Jinghong, and Lancang were significantly and positively correlated with wind speed with the correlation coefficients of 0.49, 0.59, and 0.12, respectively.”

A correction has also been made in **Results and Analysis** “Characterization of Meteorological Elements in Rubber Plantation Areas in Xishuangbanna”. This sentence previously stated:

“Temperature, sunshine duration, and average wind speed increased, whereas average relative humidity and precipitation decreased in Xishuangbanna from 1970 to 2017, as shown in **Table 4**.”

The corrected sentence appears below:

“Temperature, sunshine duration, and average wind speed increased, whereas average relative humidity and precipitation decreased in Xishuangbanna from 1970 to 2017, as shown in **Table 3**.”

A correction has been made in “Response of Climate Change and Rubber Plantation Expansion to ET_0_”, paragraph 3. This sentence previously stated:

“During 2000, it was 1.08%/10a from 1970 to 2000 and 1.97/10a from 2000 to 2017 with a significant decrease trend of 45.18% in average relative humidity.”

The corrected sentence appears below:

“During 2000, it was 1.08%/10a from 1970 to 2000 and 1.97%/10a from 2000 to 2017 with a significant decrease trend of 45.18% in average relative humidity.”

A correction has been made in “Contributions of Climate Change and Rubber Plantation Expansion to ET_0_ Trends”, paragraph 1. We will delete “which is 0.018 mm/a” from the below sentence:

“The positive contribution of rising sunshine duration and decreasing average relative humidity in Xishuangbanna caused the increase of ET_0_, which is 0.018 mm/a over the past 47 years.”

The corrected sentence:

“The positive contribution of rising sunshine duration and decreasing average relative humidity in Xishuangbanna caused the increase of ET_0_ over the past 47 years.”

A correction has been made in “Contributions of Climate Change and Rubber Plantation Expansion to ET_0_ Trends”, paragraph 2. We will delete “in 2.3.5” from the below sentence:

“Based on assessment of ET_0_ change in rubber plantation expansion in 2.3.5,”

The corrected sentence:

“Based on assessment of ET_0_ change in rubber plantation expansion,”

In the published article, we have corrected the correspondence email address in the published manuscript from “13108502978@163.com” to “shizhengtao@163.com”.

The authors apologize for these errors and state that these do not change the scientific conclusions of the article in any way. The original article has been updated.

